# Epidemiologic analysis of respiratory viral infections among Singapore military servicemen in 2016

**DOI:** 10.1186/s12879-018-3040-x

**Published:** 2018-03-12

**Authors:** Yuk-Fai Lau, Wee-Hong Victor Koh, Clement Kan, Poh-Choo Alethea Dua, Ai-Sim Elizabeth Lim, Chin-Wen Jasper Liaw, Qiu-Han Gao, Jeremiah Chng, Vernon J. Lee, Boon-Huan Tan, Jin-Phang Loh

**Affiliations:** 1 Defence Medical and Environmental Research Institute, DSO National Laboratories, Singapore, Singapore; 2 Biodefence Centre, Ministry of Defence, Singapore, Singapore

**Keywords:** Surveillance, Febrile respiratory illness, Respiratory pathogens, Military recruits

## Abstract

**Background:**

Respiratory illnesses have been identified as a significant factor leading to lost training time and morbidity among Singapore military recruits. A surveillance programme has been put in place to determine etiological agents responsible for febrile, as well as afebrile respiratory illnesses in a military camp. The goal of the study is to better understand the epidemiology of these diseases and identify potential countermeasures to protect military recruits against them.

**Methods:**

From Jan 2016 - Jan 2017, a total of 2647 respiratory cases were enrolled into the surveillance programme. The cases were further stratified into Febrile Respiratory Illness (FRI, with body temperature > 37.5 °C) or Acute Respiratory Illness (ARI, with body temperature < 37.5 °C). Nasal washes were collected and tested by multiplex PCR to detect 26 different pathogens.

**Results:**

One thousand ninety five cases (41% of total cases) met the criteria of FRI in which 932 cases (85% of FRI cases) were screened positive for at least one virus. The most common etiological agents for FRI mono-infection cases were Adenovirus E and Rhinovirus. Recruits infected with H3N2 influenza, Influenza B and Adenovirus E viruses were most likely presented as FRI cases. Notably, H3N2 influenza resulted in the greatest rise in body temperature. The remaining 1552 cases (59% of total cases) met the criteria of ARI in which 1198 cases (77% of ARI cases) were screened positive for at least one virus. The most common etiological agent for ARI mono-infection was Rhinovirus. The distribution pattern for dual infections was different for ARI and FRI cases. Maximum number of pathogens detected in a sample was five for both groups.

**Conclusion:**

Previous studies on respiratory diseases in military focused largely on FRI cases. With the expanded surveillance to ARI cases, this study allows unbiased evaluation of the impact of respiratory disease pathogens among recruits in a military environment. The results show that several pathogens have a much bigger role in causing respiratory diseases in this cohort.

**Electronic supplementary material:**

The online version of this article (10.1186/s12879-018-3040-x) contains supplementary material, which is available to authorized users.

## Background

High population density, in addition to physical and mental stress to adapt to a new environment, has been recognized to contribute to increased susceptibility of military recruits to respiratory infection [1]. To better protect the recruits and reduce loss in training time, a surveillance programme was put in place to monitor the etiological agents responsible for febrile respiratory illnesses (FRIs) among Singapore military recruits since 2009. Data from the surveillance programme showed that Influenza, Coxsackie, Rhinovirus and Adenovirus are the primary causative agents of FRIs in military camps [2, 3]. Mass vaccination of military recruits against pandemic H1N1 (A(H1N1)pdm09) in 2009 demonstrated that such a policy can reduce disease burden among recruits [4]. A subsequent study also showed that routine administration of influenza vaccine to recruits resulted in significant protection against both A(H1N1)pdm09 and influenza B, and thus, was recommended for all military personnel since [5].

While the initial focus of the surveillance programme is on etiological agents that cause febrile illnesses, some recruits were excluded from the programme as they only presented respiratory symptoms without elevation in body temperature (termed as Acute Respiratory Illnesses (ARIs)). Since late 2015, the surveillance programme was expanded to monitor the etiological agents responsible for ARIs cases as well, owing to the paucity of information on causative agents for ARI cases. Using molecular diagnostic technology, the current study aimed to evaluate the contribution of 26 different pathogens to FRI and ARI cases. This report highlights findings on both the FRI and ARI cases captured by the surveillance programme in its first year of ARI survey. Additionally, there is a focus on significant relationship between pathogens presented as co-infections.

## Methods

### Samples

The Singapore military started a respiratory diseases surveillance programme in 2009 [2]. From 4th January 2016 through 6th January 2017, servicemen who visited the medical center in a recruit training camp and presented with respiratory symptoms were enrolled in the study. Healthcare workers obtained written informed consent, administered a questionnaire and measured the body temperature using an ear thermometer. After a clinical examination on each participant, nasal wash (4 mL per nostril) was collected. The cases were stratified into two groups, namely: Acute Respiratory Illness (ARI): cases with respiratory symptoms and body temperature below 37.5 °C; and Febrile Respiratory Illness (FRI): cases with respiratory symptoms and body temperature equal to or above 37.5 °C. In addition, control samples were obtained from those reported sick without respiratory symptoms / fever. The study was reviewed and approved by DSO-SAF Institutional Review Board (IRB).

### Detection of respiratory viruses

The nasal wash samples in viral transport media were sent to a laboratory for diagnostic testing within 24 h after collection. Extraction of viral genetic materials and subsequent qualitative nucleic acid multiplex diagnostic assay were carried out according to ISO 15189 standard and have been previously described in detail [2]. Briefly, total nucleic acids were extracted from each specimen using the MagNA Pure 96 system (F. Hoffmann-La Roche Ltd) and screened for the presence of different pathogens using RT-PCR methodology with in-house developed primers and probes. For specimens that were positive for Influenza A, Adenovirus and Coronavirus, they were further subtyped using subtype-specific primers. A total of 26 bacterial and viral pathogens can be detected in this workflow. These assays were run using the 7500 Fast Real-Time PCR system (Applied Biosystems, ThermoFisher Scientific). The performance of the Real-Time PCR assays has been validated through regular participation in external proficiency programs such as Quality Control for Molecular Diagnostics (QCMD) External Quality Assessment (EQA) programs.

### Statistical methods

The significance of the difference between any two different groups was assessed by the Mann-Whitney test using Prism 5 (GraphPad Software, CA). *P* value of < 0.05 is considered statistically significant.

## Results

### Distribution of etiological agents responsible for FRI and ARI cases

A total of 2647 cases with respiratory symptoms were recruited in the study. Of these, 1095 (41%) cases were presented with a body temperature above 37.5 °C (termed as FRI cases) and in the remaining 1552 (59%) cases, the subjects had normal body temperature (termed as ARI cases). Twenty-five participants with neither fever nor respiratory illnesses, were recruited as normal control.

**Fig. 1:**
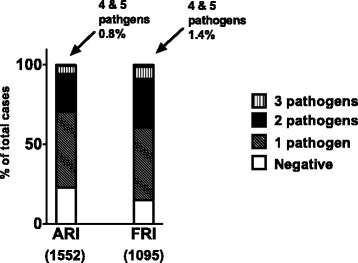
Breakdown on the number of pathogen detected from nasal wash samples

The nasal wash samples were tested for the presence of 26 different pathogens by RT-PCR methods. For the ARI cases, 738 (47.6%) and 371 (23.9%) of the tested samples were found positive for one and two pathogens respectively (Fig. 1). A total of 77 cases had three positive returns (5.0%). Eleven cases (0.7%) were found positive for four different pathogens. Finally, one ARI case was found positive for five pathogens, which is the maximum number of pathogens found in one sample for ARI group (Fig. 1). The pathogen distribution pattern was similar for the FRI cases, with 45.4%, 30.9% and 7.5% of the cases detected with one, two and three pathogens in the sample extracts respectively (Fig. 1). Twelve cases (1.1%) had four pathogens detected in the samples. Finally, three cases had five pathogens detected. The positivity rate for ARI and FRI samples was 77.2% and 85.1% respectively (Fig. 1).

The most common single pathogen found in ARI samples was Rhinovirus, which accounted for ~ 47% of the samples (Table 1). Other significant pathogens found in the mono-infection samples were *H. influenza* (19%), Adenovirus E (5.3%) and Coronavirus (6.0%, including HKU1, NL63 and OC43). Adenovirus E (27.4%), Rhinovirus (24.1%), and Influenza viruses (17.9%, H3 and influenza B) were the three most important pathogens for FRI mono-infection cases. For both FRI and ARI cases, *H. influenzae* was the most frequently found bacterial pathogen in samples.

**Table 1:** Etiological agents detected in FRI and ARI samples as mono-infection

		ARI cases		FRI cases	
	Pathogen	*N*	%	*N*	%
1.	Adenovirus	48	6.5	140	28.2
	Type B	2	0.3	3	0.6
	Type E	39	5.3	136	27.4
	Type U	7	0.9	1	0.2
2.	*B. pertussis*	4	0.5	3	0.6
3.	*C. pneumoniae*	5	0.7	0	0.0
4.	Coronavirus	49	6.6	20	4.0
	229E	4	0.5	2	0.4
	HKU1	18	2.4	10	2.0
	NL63	10	1.4	3	0.6
	OC43	16	2.2	5	1.0
	U	1	0.1	0	0.0
5.	Enterovirus	27	3.7	15	3.0
6.	*H. influenzae*	140	19.0	46	9.3
7.	hMPV	24	3.3	23	4.6
8.	Influenza A	15	2.0	36	7.2
	A(H1N1)pdm09	2	0.3	2	0.4
	H3	13	1.8	34	6.8
9.	Influenza B	24	3.3	55	11.1
10.	*M. pneumoniae*	0	0.0	0	0.0
11.	*N. meningitidis*	4	0.5	2	0.4
12.	Parainfluenza	35	4.7	31	6.2
	Type 1	0	0.0	2	0.4
	Type 2	4	0.5	5	1.0
	Type 3	18	2.4	22	4.4
	Type 4	13	1.8	2	0.4
13.	Rhinovirus	351	47.6	120	24.1
14.	RSV A	1	0.1	0	0.0
15.	RSV B	2	0.3	1	0.2
16.	*S. pneumoniae*	9	1.2	5	1.0
	Total	738	100.0	497	100.0

For the 25 normal controls, 17 of them (68%) were negative for all pathogens screened. For the remaining eight cases, five cases were found positive for *H. influenza* (2 cases as single positivity, Table 2). One case was positive with Adenovirus and two were positive for both Influenza B with Rhinovirus (Table 2).

**Table 2:** Etiological agents detected in normal controls

Pathogen found	No of cases
Adenovirus E	1
*H. influenzae*	2
Enterovirus + *H. influenzae*	2
Influenza B + Rhinovirus	2
Parainfluenza 3 + Rhinovirus + *H.influenzae*	1
Total	8

### Temporal distribution of pathogens responsible for mono-infection

**Fig. 2:**
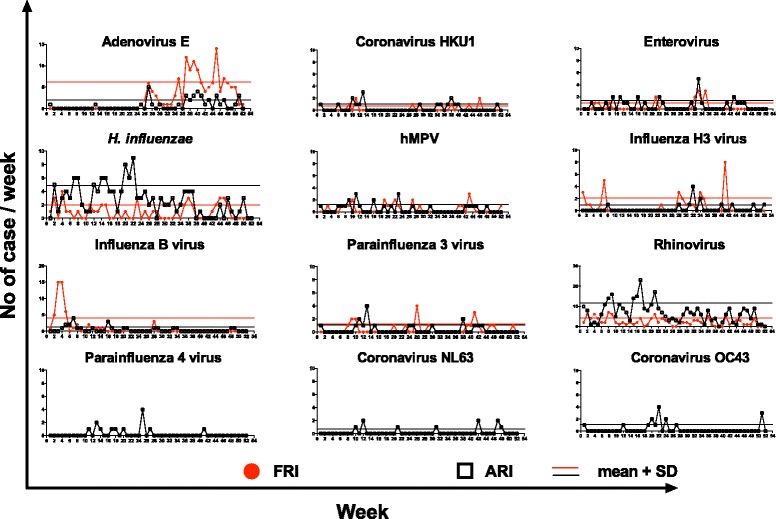
Temporal distribution of cases with mono-infection. Etiological agents with less than 10 cases in 2016 were not shown

Figure 2 summarizes the temporal distribution of cases with mono-infection.

For Adenovirus E, the majority of the Adenovirus E cases occurred in the second half of 2016. From Week 36 to Week 52, a total of 106 of FRI (red line) and 28 ARI (black line) cases were reported. Eight recruits were diagnosed with pneumonia. For Influenza H3 virus, there were three main peaks for FRI cases in 2016. The first one occurred between Week 5 to 8. The second peak occurred between Week 23 to Week 35 in which 14 cases were reported. The last peak consisting of 8 cases, occurred between Week 40-42. There were very few cases of influenza H3 infection without fever symptom in 2016. For Influenza B virus, there was one main peak for FRI cases in 2016 which involved 43 cases between Week 1-6. There were only sporadic FRI cases reported for the rest of the year. Influenza B was not a significant etiological agent for ARI cases. FRI and ARI cases caused by Enterovirus, hMPV, Parainfluenza 3 virus and Rhinovirus were reported throughout the year, with no distinct seasonality. In general, most cases caused by these pathogens were presented as ARI cases. Finally, for Parainfluenza 4 virus, Coronaviruses NL63 and OC43, sporadic cases with no fever symptom were reported throughout the year.

### Rise in body temperature caused by different pathogens in mono-infections

**Fig. 3:**
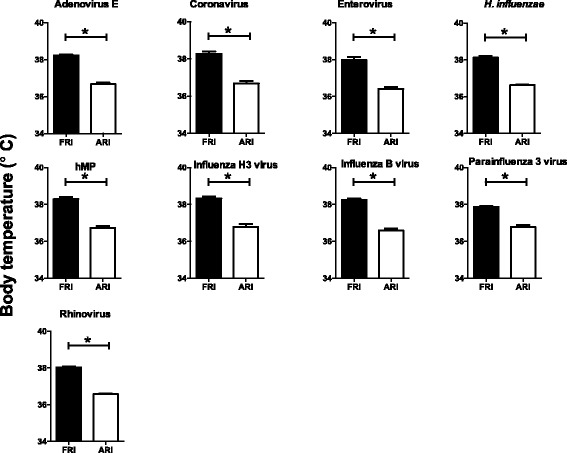
Rise in body temperature based on etiological agents with mono-infection

**Fig. 4:**
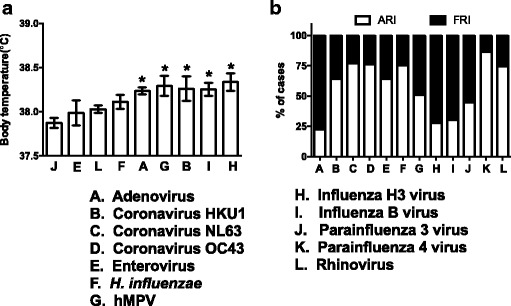
**a** Average body temperature of FRI mono-infection. **b** FRI and ARI distribution for each etiological agent. Only those with more than 20 cases detected in 2016 were shown

All the FRI and ARI cases with one pathogen detected were further stratified into groups; based on the pathogen detected in the samples. The mean and standard error of the body temperature of each group was plotted (Fig. 3) and the significance of the difference between the FRI and ARI groups was determined. All the FRI cases, irrespective of the etiological agent involved, had a significantly higher body temperature than the ARI cases (*p* < 0.05, Fig. 3). Notably, there was a relationship between the level of body temperature increment and the etiological agent detected. For example, those infected with Parainfluenza 3 virus had the least elevation in body temperature (average = 37.9 °C) with respect to those infected with Influenza H3 virus where the average body temperature was 38.3 °C. Using Parainfluenza 3 virus as a reference, the body temperature elevation was significantly higher in those infected with Adenovirus E, hMPV, Coronavirus HKU, Influenza B and Influenza H3 virus (p < 0.05, Fig. 4a). Recruits infected with Adenovirus, Influenza H3 and Influenza B viruses were more likely to be presented as FRI cases, whereas those infected with Coronaviruses, Enterovirus, Parainfluenza 4 and Rhinovirus were more likely to be ARI cases (Fig. 4b).

### *H. influenzae* was the most frequently observed pathogen in dual infections

A total of 371 ARI (23.9%) and 338 FRI (30.9%) cases had two different pathogens detected in the samples. For ARI cases, the most commonly observed combination was Rhinovirus with *H. influenzae* (Table 3, 129 cases)*,* consist of 34.8% dual infection cases. In addition, for those positive for *H. influenzae* cases, Enterovirus (16 cases) and Adenovirus E (14 cases) were frequently reported together (Table 3).

**Table 3:** The combinations of etiological agents most commonly detected together in FRI and ARI cases

	No. of case (%^a^)
ARI	
Rhinovirus + *H. influenzae*	129 (34.8%)
Enterovirus + *H. influenzae*	16 (4.3%)
Adenovirus E + *H. influenzae*	14 (3.8%)
FRI	
Adenovirus E + *H. influenzae*	62 (18.3%)
*H. influenzae* + Rhinovirus	41 (12.1%)
Adenovirus E + Rhinovirus	36 (10.7%)

^a^ % of all dual infections detected

For FRI cases with dual infections, *H. influenzae* was the most frequently observed bacterial pathogen, with 62 cases associated with Adenovirus E (18.3%) and 41 cases with Rhinovirus (12.1%). Other significant combinations include Adenovirus E with Rhinovirus (36 cases, 10.7%). The detailed distribution pattern of the combinations found in 2016 as dual infections can be found in Additional file 1.

**Fig. 5:**
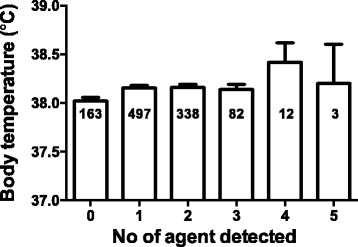
Average body temperature of recruits stratified based on the number of pathogens detected in their samples

We also examined if cases with dual pathogen detected have higher body temperature elevation compared to the mono-infection. The result shows that the two groups had similar degree of evaluation in body temperature and no significant statistical difference (Fig. 5, *p* = 0.5992).

### Pathogen distribution in cases with three or more agents detected

A total of 77 ARI (5.0%) and 82 FRI (7.5%) cases were detected with three pathogens in the samples. The most common triple combinations detected in ARI cases were Rhinovirus, *H. influenzae* with Influenza B virus (6 cases, 7.8%) and Rhinovirus, *H. influenzae* with Parainfluenza 3 virus (5 cases, 6.5%). For FRI cases, Adenovirus E, *H. influenzae* and Rhinovirus was the most commonly found combination (15 cases), representing 18.3% of the triple infection cases. The temperature elevation observed in these cases was similar to those caused by mono or dual pathogen (Fig. 5, *p* = 0.9650).

Eleven ARI cases (0.7%) had 4 pathogens detected in the samples and the combinations can be found in Additional file 2. One ARI case had five pathogens detected in the sample (Coronavirus HKU1, *H. influenzae*, Influenza B, Parainfluenza 2 and Rhinovirus), representing the highest number of pathogens detected in one sample.

Twelve (1.1%) and three (0.3%) FRI samples had four and five pathogens detected. The combinations can be found in Additional file 2. Although these cases had a higher average body temperature, the differences did not reach statistical significance (Fig. 5, *p* = 0.2065, 0.3547, 0.9328).

### Other significant pathogens detected in 2016

A total of 17 cases were found positive for *B. pertussis* in 2016. Four cases presented as mono-infection for ARI and three cases for FRI category. The remaining 10 cases, *B. pertussis* was detected among the multiple pathogens, consisted of five cases for each of the ARI and FRI category. For *N. meningitides*, 27 cases were found positive in 2016. Six cases of mono-infection, make up of four ARI and two FRI cases. The remaining cases were with multiple pathogens detected, consisted of eight ARI and 13 FRI cases respectively. Finally, one case of ARI was found positive for Bocavirus. It was a co-infection case, with 3 additional pathogens (Coronavirus OC43, Enterovirus, *H. influenzae*) detected together.

## Discussion

In a 2006 study, Influenza A and B viruses were reported as important etiological agents for FRI cases among the recruits in Singapore [3]. Thirty-six percent of FRI cases were positive for Influenza A and B viruses. Numerous outbreaks of Influenza virus in military camps have been reported [6, 7], reviewed in [1]) and one study showed that the attack rate of influenza infection for cadet was four times higher than that observed in high rank officers (31.4% v.s. 7.7%) [6]. To mitigate the burden of Influenza illness among recruits in Singapore, routine influenza vaccine has been given to recruits since 2010. Ho et al. reported that the strategy was effective in reducing the risk of A(H1N1)pdm09 virus and Influenza B infection, but not for Influenza A (H3N2) [5]. Data presented in this study are consistent with Ho et al. [5] -- A(H1N1)pdm09 virus was no longer a significant FRI etiological agent in 2016. Apart from a small scale outbreak of Influenza B infections in early 2016, there were only sporadic Influenza B cases reported for the rest of the year. The outbreak coincided with the prevalence of Influenza B in the community during that period. Similarly, during the H3 outbreak which peaked between Week 28-32, H3N2 Influenza A virus was the dominant circulating strain (82.6%-88.7%) in the community [8]. Although recruits had restricted access to the community during the training period, other military personnel, that are not required to reside in camps at night, could carry the circulating strains from the community back to the camps to start the outbreaks. Given the dramatic reduction in FRI cases caused by Influenza A and B viruses, influenza vaccination should be continue to be given to all recruits immediately after enlistment.

With 136 FRI and 39 ARI cases caused by Adenovirus E in 2016, it has become a significant respiratory pathogen in the training camp. Another similar Adenovirus outbreak in Singapore military training camp was reported in 2005. In the 2005 outbreak, 30 recruits were affected, and body ache, headache and nasal congestion were the most frequently reported symptoms. Adenovirus 11a was subsequently identified as the causative agent of that outbreak [9]. Adenovirus type 4, the only member in the species E, was also detected among military personnel with FRI in another study conducted between 2011 and 2012 [10]. Currently, addition experiments are underway to better characterize the 2016 isolates and whether there are any new mutations in the viral genome. While the majority of adenoviral infections cause mild symptoms and are self-limiting, cases of fatality have been reported in other military forces [11–13]. In 2011, an outbreak of Adenovirus Type 7 was reported in a Malaysian police training centre, affecting 851 trainees, with 100 being hospitalized and 3 deaths [13]. More recently, HDAdV-55 was found to be responsible for an outbreak among Korean military personnel with 1 case of fatality [11]. Adenovirus B was also found to be responsible for febrile respiratory illness in a military training camp in China [14]. Serological surveillance on US military basic trainees showed that 66% and 73% of trainees were susceptible to Adenovirus Type 4 and 7 [15]. Similar trend was observed by Russell et al. [16]. Viral genetic material and virus could be recovered from various environmental samples, such as lockers, rifles, pillows, highlighting the possibility of transmission among the recruits. [16]. For countermeasures, live oral vaccines against Types 4 and 7 have been given to the US army recruits from 1971 to 1999. Following the cessation of the vaccination programme between 1999 and 2011, there was a resurgence of adenovirus outbreaks [17]. The vaccination programme was re-introduced in 2011 and resulted in a dramatic reduction in cases, demonstrating the effectiveness of vaccination in controlling adenovirus respiratory illnesses among this high risk group [18].

Pertusis is another vaccine-preventable disease and pertussis vaccination has been given to all newborns in Singapore since 1957 [19] and sero-prevalence of pertussis antibodies in the adult population of Singapore (18-45 years) was 97% in 2002 [20]. In 2015, there was a total of 56 local cases of pertussis reported in Singapore, a rise from the 19 cases in 2014. As vaccine-induced antibodies begin to wan significantly 4 years after the last dose [21], a booster could be beneficial for optimal protection of the recruits against this highly contagious bacterial disease. Such benefit has recently been reported from a study involving military conscripts from Finland [22].

For 2016, the most common etiological agents reported in FRI mono-infections were Adenovirus E (136 cases, 27.4%), Rhinovirus (120 cases, 24.1%), Influenza B virus (55 cases, 11%) and Influenza A H3 virus (34 cases, 6.8%). The absolute number of FRI cases caused by Influenza viruses has reduced significantly in 2016, likely owing to the mass vaccination of recruits against seasonal influenza (485 cases in 2006 [3] and 1007 cases (2009-2012) [2] and 91 cases in 2016 (current study)). In contrast, FRI cases caused by Adenovirus have been on the rise –(5 cases in 2006, 100 cases between 2009 and 2012) and 136 cases in 2016 alone). On the other hand, Rhinovirus has been an important etiological agent for FRI cases for the past 7 years (581 cases for 2009-2012 and 162 cases for 2016). While Rhinovirus and Adenovirus are common etiological agents responsible for FRI cases reported in studies from other military recruit camps, some agents are unique only to a particular study. For example, Levy et al. reported that Rhinovirus, Coronavirus 229 and Parainfluenza 4 were the main FRI etiological agents for recruits of the Royal Thai Army basic training classes [23]. No cases for Adenovirus or Influenza A virus were reported during the study period. A high isolation rate of Adenovirus was reported in a study in Korea [24]. However, another study performed in Korea, found that Rhinovirus, Enterovirus and Coronavirus OC43 were commonly found in recruits with respiratory symptom [25]. Using paired samples, Eslami-Nejad et al. showed an increase in *N. meningitidis* carrier rate when recruits in Iran completed their basic training course [26]. Similarly, bacterial infections caused by *B. pertussis*, *Chlamydia pneumoniae*, *Mycoplasma pneumoniae* could have occurred among the military service members deployed to Afghanistan [27]. Clearly, multiple factors like the study population, the seasonality of the study period, the type of samples collected, the geographical location of the study, the level of herd immunity of the locals and the exposure of novel pathogens in a new operational environment -- all could influence the type and variety of etiological agents to be found and reported in these studies.

FRI cases have been the main focus of our previous studies and they have provided valuable insights on the importance of various pathogens responsible for febrile respiratory illnesses in military recruits in Singapore [2, 3]. They also led to the implementation of effective countermeasures for better protection of the recruits. The current study expanded from the previous studies by including respiratory cases without fever, so as to gain a more comprehensive understanding on the etiological agents circulating among the recruits. Data from this expanded study revealed that while Rhinovirus has been an important etiological agent for FRI cases, it plays an even bigger role for ARI cases. Similarly, most of the cases caused by Parainfluenza 4 were presented as ARI cases. Additionally, the most common ARI agents involved in dual infections were Rhinovirus and *H. influenzae, while* Adenovirus E and *H. influenzae* were commonly detected together in FRI cases. In view of the new perspectives and insights into respiratory infections among recruits, gleaned from our findings, a surveillance programme with a wider scope should be continued.

There are limitations to the present study. Firstly, some bacterial pathogens, such as *H. influenzae* and *N. meningitidis,* are present as commensals in certain individuals. Detection of Rhinoviruses from asymptomatic individuals has been reported [28], therefore, positive detection in the sample may not indicate that they are the etiological agents responsible for the disease. Similarly, for samples with multiple pathogens detected, it is not feasible to determine the respective contribution of each pathogen to the disease. Further investigations are needed to determine the clinical relevance of co-infections and their management. Secondly, our study was limited to pathogen identification, without consideration of the pathogen load in the sample. Thirdly, the study focused mainly on respiratory symptoms and did not track other serious conditions such as meningitis. Fourthly, due to the large number of samples, pathogen isolation from samples could not be done for further verification of the findings. In addition, the sample size of the control samples was small. Fifthly, since the classification of ARI and FRI is based on a single temperature measurement, we could not rule out the possibility that some ARI cases could have become FRI cases subsequently. This limitation could have led to an under-estimation of the number of FRI cases. Lastly, the study predominantly involved young adult males in a semi-closed military setting, hence the findings reported in this study might not be applicable to the adult general population.

## Conclusion

The current study describes the etiological agents responsible for FRI and ARI among the military recruits in the tropical urban setting. By studying both FRI and ARI cases in the same study period, it allowed a more complete picture of the respiratory pathogens circulating in the training camp. It also provides us with an unbiased assessment of the overall impact of a particular pathogen in causing respiratory illnesses. This is exemplified with the observation that Rhinovirus, apart from being an important FRI agent, it plays an even bigger role for ARI cases. With the rise in FRI cases by Adenovirus infection, apart from promoting personal hygiene awareness, it may be worthwhile to evaluate the risk-to-benefit ratio for introducing adenovirus vaccination to the recruits.

## Additional files

Additional file 1: Detailed distribution pattern of the combinations found in 2016 as dual infections. (PDF 116 kb)

Additional file 2: FRI and ARI samples with 4 or 5 pathogens detected. (PDF 30 kb)

## Abbreviations

ARI: Acute respiratory illness
FRI: Febrile respiratory illness
hMPV: Human Metapneumovirus
Real-time PCR: Real-time polymerase chain reaction
