# Supplementary material for: Epidemiologic analysis of respiratory viral infections among Singapore military servicemen in 2016
Source: BMC Infect Dis. 2018 Mar 12;18:123. doi: 10.1186/s12879-018-3040-x (PMC5848554; doi:10.1186/s12879-018-3040-x)
Supplement: Supplementary file 1 — Detailed distribution pattern of the combinations found in 2016 as dual infections. (PDF 116 kb) [file 12879_2018_3040_MOESM1_ESM.pdf]

	Adenovirus B	Adenovirus E	Adenovirus U	B. pertussis	C. pneumoniae	Coronavirus 229E	Coronavirus HKU1	Coronavirus NL63	Coronavirus OC43	Coronavirus U	Enterovirus	H. influenzae	hMPV	Influenza A H1N1 (pdm 09) viru	Influenza A H3 virus	Influenza B virus	M. pneumoniae	N. meningitidis	Parainfluenza 1 virus	Parainfluenza 2 virus	Parainfluenza 3 virus	Parainfluenza 4 virus	Rhinovirus	RSV A	RSV B	S. pneumoniae
Adenovirus B											1															
Adenovirus E							1		1		4	14	3			1		1		1	1		10			
Adenovirus U				1			1				1	6											7			
B. pertussis												1										1	1			
C. pneumoniae											1												1			
Coronavirus 229E												1											2			
Coronavirus HKU1											3	13						1					7			
Coronavirus NL63																1						1	3			1
Coronavirus OC43												6				1						1	6	1		
Coronavirus U																							1			
Enterovirus												16	2			1		1			1	1				1
H. influenzae													11			3		1		2	13	9	129	3		3
hMPV																		1			3	3	5			
Influenza A H1N1 (pdm 09) virus																										
Influenza A H3 virus																										1
Influenza B virus																							12			
M. pneumoniae																										
N. meningitidis																						1	3			
Parainfluenza 1 virus																							1			
Parainfluenza 2 virus																							3			1
Parainfluenza 3 virus																							8			1
Parainfluenza 4 virus																							7			1
Rhinovirus																								1		14
RSV A																										
RSV B																										
S. pneumoniae																										

	Adenovirus B	Adenovirus E	Adenovirus U	B. pertussis	C. pneumoniae	Coronavirus 229E	Coronavirus HKU1	Coronavirus NL63	Coronavirus OC43	Coronavirus U	Enterovirus	H. influenzae	hMPV	Influenza A H1N1 (pdm 09) virus	Influenza A H3 virus	Influenza B virus	M. pneumoniae	N. meningitidis	Parainfluenza 1 virus	Parainfluenza 2 virus	Parainfluenza 3 virus	Parainfluenza 4 virus	Rhinovirus	RSV A	RSV B	S. pneumoniae
Adenovirus B		1						1				1														
Adenovirus E							12	3	4	1	12	62	6			3	1	1		1	5	2	36			3
Adenovirus U							1									1					1	1	3			
B. pertussis										1											1		2			
C. pneumoniae																										
Coronavirus 229E							1																1			
Coronavirus HKU1											1	1	2		3						1		4		1	
Coronavirus NL63												3											1			
Coronavirus OC43												6		1				1			1		1			
Coronavirus U																										
Enterovirus												6				2				1	1					1
H. influenzae													10	1	3	11				2	6	4	41	1	1	1
hMPV															1								5			2
Influenza A H1N1 (pdm 09) virus																							2			
Influenza A H3 virus																							5			2
Influenza B virus																					1		12			4
M. pneumoniae																							0			
N. meningitidis																							1			
Parainfluenza 1 virus																										
Parainfluenza 2 virus																							2			
Parainfluenza 3 virus																							7			1
Parainfluenza 4 virus																										
Rhinovirus																								1	1	4
RSV A																										
RSV B																										
S. pneumoniae																										
