# Supplementary material for: Epidemiologic analysis of respiratory viral infections among Singapore military servicemen in 2016
Source: BMC Infect Dis. 2018 Mar 12;18:123. doi: 10.1186/s12879-018-3040-x (PMC5848554; doi:10.1186/s12879-018-3040-x)
Supplement: Supplementary file 2 — FRI and ARI samples with 4 or 5 pathogens detected. (PDF 30 kb) [file 12879_2018_3040_MOESM2_ESM.pdf]

No	ARI samples with 4 pathogens detected
1	Adenovirus E + Coronavirus HKU1 + H. influenzae + Parainfluenza 3
2	Adenovirus E + Enterovirus + Influenza B + H. influenzae
3	Adenovirus U + H. influenzae + Influenza B + Rhinovirus
4	Bocavirus + Coronavirus OC43 + Enterovirus + H. influenzae
5	H. influenzae + hMPV + Parainfluenza 2 + Rhinovirus
6	Enterovirus + H. influenzae + Parainfluenza 3 + RSV A
7	H. influenzae + Influenza B + Parainfluenza 4 + Rhinovirus
8	hMPV + H. influenzae + Parainfluenza 3 + Rhinovirus
9	hMPV + H. influenzae + Parainfluenza 4 + Rhinovirus
10	H. influenzae + Influenza B + Rhinovirus + S. pneumoniae
11	hMPV + Parainfluenza 4 + Rhinovirus + RSV B

No	ARI samples with 5 pathogens detected
1	Coronavirus HKU1 + H. influenzae + Influenza B + Parainfluenza 2 + Rhinovirus

No	FRI samples with 4 pathogens detected
1	Adenovirus E + Coronavirus HKU1 + H. influenzae + Rhinovirus
2	Adenovirus E + Coronavirus HKU1 + H. influenzae + hMPV
3	Adenovirus E + Coronavirus OC43 + Enterovirus + H. influenzae
4	Adenovirus E + Coronavirus OC43 + H. influenzae + Rhinovirus
5	Adenovirus E + Enterovirus + H. influenzae + hMPV
6	Adenovirus E + H. influenzae + hMPV + Rhinovirus
7	Adenovirus E + H. influenzae + Rhinovirus + S. pneumoniae
8	Adenovirus E + H. influenzae + hMPV + Parainfluenza 3
9	B. pertussis + H. influenzae + hMPV + Influenza B
10	Coronavirus HKU1 + Parainfluenza 3 + Rhinovirus + S. pneumoniae
11	Coronavirus OC43 + H. influenzae + Parainfluenza 3 + Rhinovirus
12	Coronavirus OC43 + N. meningitidis + Parainfluenza 3 + S. pneumoniae

No	FRI samples with 5 pathogens detected
1	Adenovirus E + Coronavirus HKU1 + H. influenzae + Parainfluenza 3 + Rhinovirus
2	Adenovirus E + H. influenzae + N. meningitidis + Parainfluenza 4 +Rhinovirus
3	Coronavirus OC43 + H. influenzae + Influenza B + Rhinovirus + S. pneumoniae

Supplementary Table 2
